# Why are clams steamed with wine in Mediterranean cuisine?

**DOI:** 10.1038/s41538-024-00279-6

**Published:** 2024-07-11

**Authors:** Fangzhou He, Zhaoshuo Yu, Sihao Luo, Xiangyu Meng, Leying Wang, Xuanlu Jin, Zongke Huang, Yue Zhang, Peishan Deng, Weng Kung Peng, Lijing Ke, Huiqin Wang, Jianwu Zhou, Patrick Wall, Pingfan Rao

**Affiliations:** 1 SIBS-Zhejiang Gongshang University Joint Centre for Food and Nutrition Sciences, Zhejiang Gongshang University, Hangzhou, 310012 China; 2 Songshan Lake Materials Laboratory, University Innovation Park, Dongguan, 523-808 China; 3 National Nutrition Surveillance Centre, University College Dublin, Belfield, Dublin 4, Ireland; 4 UCD Institute of Food and Health, University College Dublin, Belfield, Dublin 4, Ireland; 5 School of Food Science and Nutrition, University of Leeds, Leeds, LS2 9JT UK; 6 College of Food and Bioengineering, Fujian Polytechnic Normal University, Fuqing, Fujian, 310300 China

**Keywords:** Nanoparticles, Biochemistry

## Abstract

Wine is renowned for its rich content of polyphenols, including resveratrol (Res), known for their health promoting properties. Steamed clam with wine, a popular Mediterranean delicacy that highlights the role of wine as a key ingredient. However, despite these benefits, resveratrol’s low bioavailability poses challenges. Could the process of steaming together with clam alter the digestive fate of resveratrol from wine? This study explores the potential of proteoglycan-based nanoparticles from freshwater clam (CFNPs) as a delivery vehicle for enhancing the stability and bioavailability of resveratrol, compared with wine and free Res’ solution, aiming to elucidate mechanisms facilitating Res’ absorption. The results demonstrated that CFNPs can effectively encapsulate Res with an efficiency over 70%, leading to a uniform particle size of 70.5±0.1 nm (PDI < 0.2). Resveratrol loaded in CFNPs (CFNPs-Res) exhibited an improved antioxidant stability under various conditions, retaining over 90% of antioxidant capacity after three-day storage at room temperature. The controlled-release profile of Res loaded in CFNPs fits both first and Higuchi order kinetics and was more desirable than that of wine and the free Res. Examined by the simulated gastrointestinal digestion, CFNPs-Res showed a significantly higher bioaccessibility and antioxidant retention compared to free Res and the wines. The discovery and use of food derived nanoparticles to carry micronutrients and antioxidants could lead to a shift in functional food design and nutritional advice, advocating much more attention on these entities over solely conventional molecules.

## Introduction

The Mediterranean diet stands out as a popular and healthy dietary pattern emphasising a rich intake of polyphenol-rich foods^1^. A classic dish within this regimen, Steamed Clams with Wine, has garnered global acclaim among food enthusiasts. Beyond merely enhancing the dish’s flavour, wine, a key component, is renowned for its potential health benefits owing to its high resveratrol content, known for its powerful antioxidant properties^2^. Resveratrol has potential health benefits, such as anti-inflammatory and antioxidant properties, and may help to reduce the risk of many non-communicable diseases, such as cardiovascular disease^3,4^. However, the challenges arising from resveratrol’s insolubility and instability restricted its bioavailability and efficacy^5^.

One solution to this problem is the use of nano-carriers, which are small particles, less than 100 nanometres in size, that can encapsulate and protect the antioxidants, improve its solubility, and enhance its bioavailability^6^. Various macromolecules such as proteins and polysaccharides, as well as molecules like polyvalent metals and amino acids, have served as the foundation for the design, fabrication, and utilisation of diverse nano-carriers. These carriers are tailored to deliver concentrated antioxidants effectively to targeted parts of the body, including the skin, gastrointestinal tract, and brain, with the aim of achieving discernible effects^7^. For instance, milk-derived proteins have been shown to interact with flavonoids and polyphenols like curcumin and quercetin, leading to their improved stability and altered digestive fate in vitro and in vivo through the formation of complex micro/nanostructures^8^.

In addition to the above delivery methods, the focus has started shifting towards native nanoparticles derived from food for their advantages of being affordable, stable, green, and widely available. Cooking food can produce native colloidal particles that fall within the nanometre size range and render bioactive ingredients and micronutrients more absorbable^9^. The article “*The power of soups: Super-hero or team-work?*” emphasises that food processing goes beyond simple cooking and preservation; it can actually lead to the creation of well-organised micro/nano-structures^10^ rather than the irregular aggregates. During food processing, a complex interplay of physical, biological, and chemical reactions takes place. These reactions precisely involve a trilogy: (1) destruction of cells, (2) the migration of subcellular structures within the ingredients into the liquid phase, (3) the generation of new components and the self-assembly of micro/nanostructures through the formation of covalent bonds and secondary bonds.

Food nanoparticles resulting from processing alter the physicochemical properties of micronutrients, enhancing stability and offering potential as efficient nutrition delivery systems. For example, a gradually glycosylated proteins of black tea resulting from the fermentation process imparts the tea infusion a pleasing amber colour while forming a nano-carrier skeleton that plays a crucial role in the stable presence of ample tea polyphenols^11,12^. A stable colloidal system was formed in lamb soup as a function of heat treatment and time wherein the secondary and tertiary structures of colloidal particles underwent significant changes^13^. Rice vinegar contains nanoparticles with diverse morphological characteristics and high amounts of polyphenols and flavonoids, exhibiting excellent intracellular and cellular antioxidant capacity even after years of storage^14^.

Previous studies have reported the existence of abundant proteoglycan-based nanoparticles, also known as clam nanoparticles (CFNPs), in the soup of the freshwater clam (*Corbicula fluminea*), which are generated during the heat treatment of clams^15,16^. In subsequent research, micelles or nanoparticles exhibiting analogous nanostructures based on proteins and polysaccharides have been extensively identified in more than ten varieties of shellfish soup, including Mediterranean mussel and Pacific oyster. This reveals the prevalent occurrence of macromolecular self-assembly in thermal processing of shellfish. CFNPs have exhibited the ability to carry both water-soluble compounds, such as taurine and ornithine, as well as fat-soluble compounds, such as phytosterols, while maintaining their structural stability under conditions of high temperature and extreme acidity^17^. Remarkably, a significant quantity of nanoparticles can be also isolated from distillate of shellfish, a typical by-product of shellfish processing, containing protein, polysaccharides, and functional lipids like polyunsaturated fatty acids^18,19^. Therefore, CFNPs stand as a good example of food derived nanocarriers that are easy-to-prepare, efficient and resilient.

Our hypothesis is that CFNPs can carry resveratrol from wine and improve its subsequent stability and bioavailability. This study delves into the binding kinetics and mechanisms between resveratrol and CFNPs, exploring their colloidal properties and stability during room temperature storage. A comparative analysis was conducted among CFNPs-bound resveratrol, free resveratrol, and resveratrol sourced from specific wines. Additionally, the release kinetics of resveratrol, its bioaccessibility, and antioxidant stability under simulated digestion were assessed. This study aims to establish a model of the interaction between food nanoparticles and resveratrol, elucidate the capacity and feasibility of nutrient delivery system within the clam soup, while providing an unique microstructural perspective to explore the nutritional value of the popular Mediterranean dish, Steamed Clams with Wine.

## Results and discussion

### Isolation of CFNPs from soup and characteristics of resveratrol loaded CFNPs

**Fig. 1:**
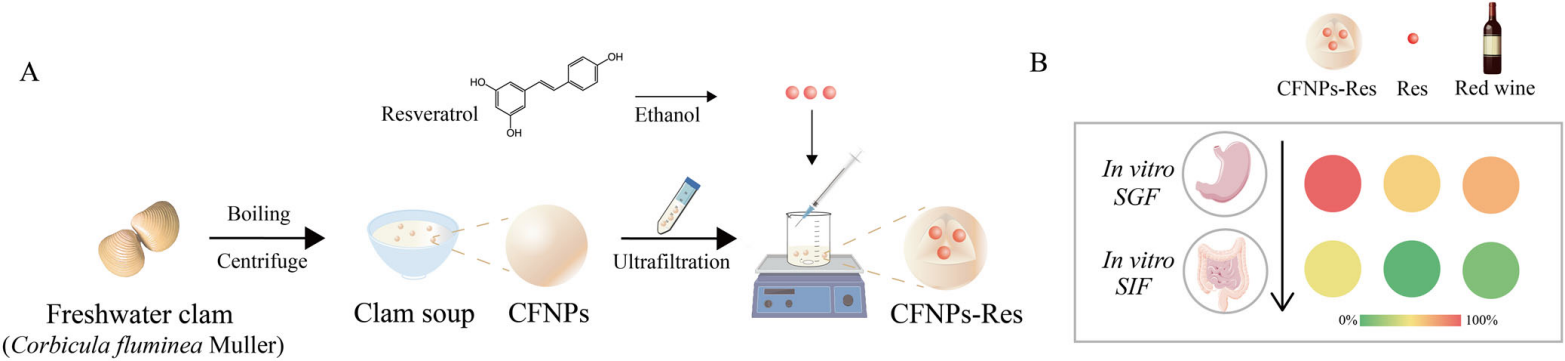
Experiment design. **A** Freshwater clam soup was boiled with pure water in a 1:1 (w/v) ratio and then centrifuged to obtain the supernatant. The CFNPs were isolated from the supernatant using ultrafiltration (100 kDa). Subsequently, Res was added dropwise to the CFNPs solution using a magnetic stirrer to form the composite CFNPs-Res. The procedure was performed with careful attention to detail and in adherence to established protocols for the preparation of these materials. **B** The retention rate of antioxidation capacity of Res, CFNPs-Res and wines during in vitro simulated digestion was evaluated, and the results were presented as a heat map.

**Fig. 2:**
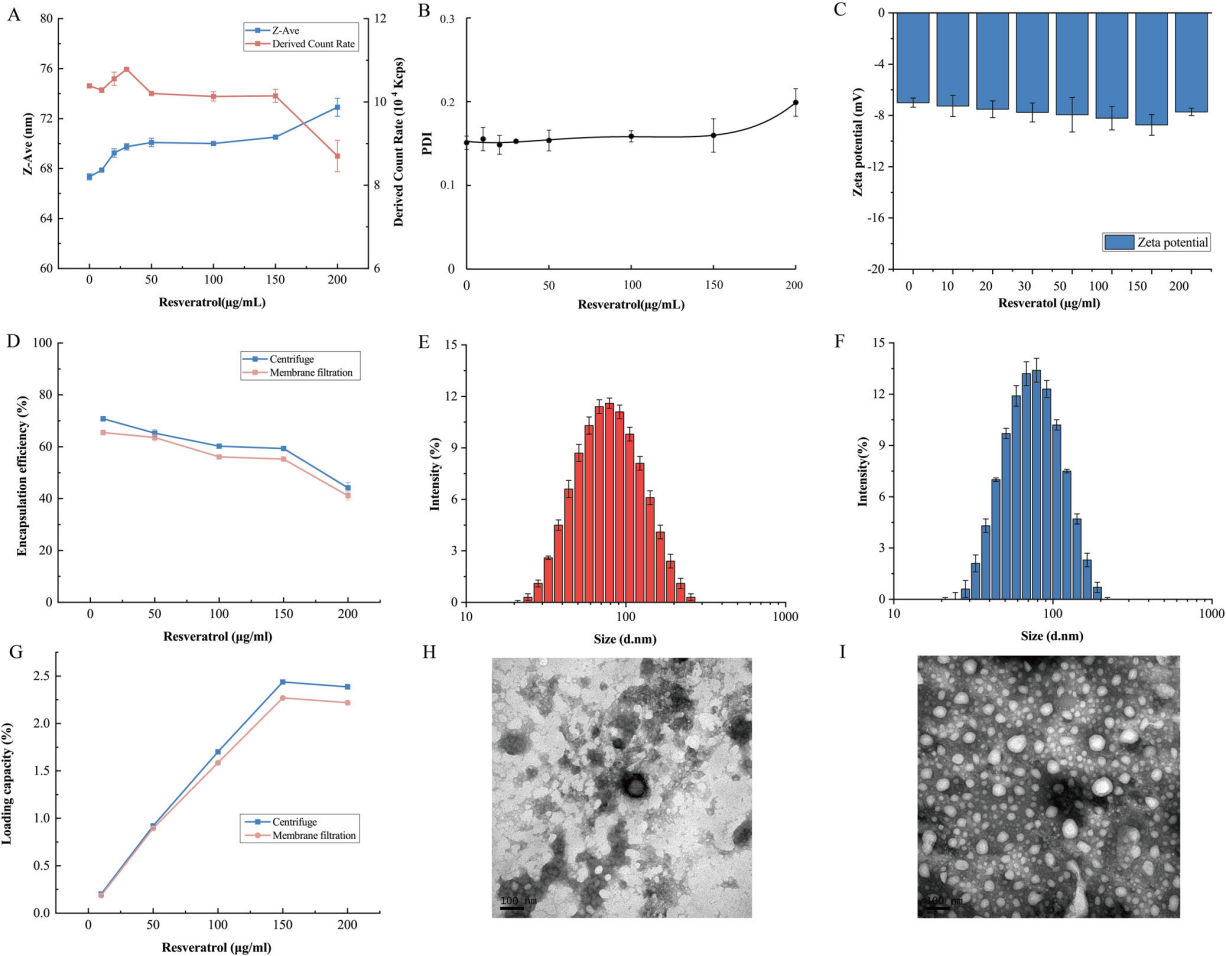
Characteristics of CFNPs and CFNPs-Res. **A** The average particle size and intensity of light scattering of CFNPs-Res under various Res concentrations ranging from 10 to 200 μg/mL. **B** PDI, (**C**)ζ-potential, (**D**, **G**) EE and LC of CFNPs-Res under various Res concentrations ranging from 10 to 200 μg/mL. **E**, **F** The size distribution of CFNPs and 150 μg/mL CFNPs-Res. **H**, **I** TEM images of CFNPs and 150 μg/mL CFNPs-Res. Error bars refer to standard deviation.

As depicted in Fig. 1, this article mainly consisted of three parts, including the harvesting CFNPs from freshwater clam soup, the interaction of CFNPs with resveratrol, and the evaluation of the antioxidant stability of CFNPs-Res. The size of CFNPs isolated from the soup was 67.3 ± 0.2 nm with a ζ-potential of −6.9 ± 0.3 mV determined by DLS. The effects of resveratrol concentration (10–200 μg/mL, based on the final concentration) on the formation of resveratrol-loaded nanoparticles were evaluated in terms of visual observation, intensity of light scattering, particle size, ζ-potential, polydispersity index (PDI), and encapsulation efficiency and loading capacity, as depicted in Fig. 2. Specifically, a significant increase in the average size of CFNPs- resveratrol was observed with an increasing Res concentration within the range of 10–30 μg/mL, followed by a relatively stable size until the resveratrol concentration reached 150 μg/mL, where a remarkable increase in size was then observed. The intensity of light scattering exhibited fluctuations between 10 and 50 μg/mL of resveratrol concentration and remained stable until a sharp decrease was observed at 150 μg/mL of resveratrol concentration (Fig. 2A). This suggests that the interaction of CFNPs and resveratrol was highly dependent on the concentration of resveratrol with a critical concentration of 150 μg/mL. At the critical point, the size of CFNPs-Res was found to be 70.5 ± 0.07 nm, which was slightly larger than that of CFNPs (67.3 ± 0.2 nm). Both CFNPs and CFNPs-Res had a unimodal size distribution (Fig. 2E, F).

The PDI, commonly used to evaluate size heterogeneity, remained constant (below 0.2) until the resveratrol concentration reached 150 μg/mL, after which it exhibited a substantial increase (Fig. 2B). Typically, a PDI value below 0.2 indicates the presence of a unimodal size distribution, which is consistent with the findings on particle size. These results validate the occurrence of size disorder upon surpassing the critical resveratrol concentration.

ζ-potential generally plays an important role in evaluating the stability of nanoparticles as it is determined by the surface charge that causes electrostatic repulsive interactions between particles. As shown in Fig. 2C, increasing resveratrol concentration led to a slight increase in absolute value of ζ-potential from −6.9 to −8.7 mV, indicating the interaction of CFNPs and resveratrol might contribute to their stability. These results prove that resveratrol did not significantly change the ζ-potential of nanoparticles, which was consistent with the previous research by Neves et al.^20^.

The morphology of CFNPs and CFNPs-Res was investigated by TEM, respectively (Fig. 2H, I). Both CFNPs and CFNPs-Res were found to exhibit approximately spherical shapes with a smooth surface. However, an evident distinction between the two was observed. CFNPs-Res exhibited a characteristic corona shell enveloping its surface, suggesting the successful binding of resveratrol into the CFNPs and the formation of a complex nanostructure. Besides, the size of CFNPs and CFNPs-Res determined by TEM was consistent with the results obtained from the DLS determination. Above a resveratrol concentration of 150 μg/mL, an unusual phenomenon was observed.

For the single particle-dominated system, it is common for the light scattering intensity to be proportional to both the number and size of particles. However, contrary to expectations, as particle size increased, the light scattering intensity significantly decreased. Interestingly, despite the increase in size, there was no visible precipitation, indicating that the particles remained relatively stable, and their number did not change significantly. Therefore, this phenomenon can be attributed to the presence of additional particles in the system when the resveratrol concentration exceeded the critical threshold.

Previous studies^21^ have indicated that due to its poor water solubility, free resveratrol is likely to self-assemble into micelle particles larger than 100 nanometres or even micrometres in the water solution when the concentration exceeds the maximum loading capacity of nanocarriers. This can modify the particle size distribution and an increase in the size of CFNPs-Res. Moreover, ample micelles probably result in deviations in light scattering intensity, such as multiple light scattering, where laser scatters repeatedly between multiple particles^22^, leading to a lower intensity for CFNPs-Res. These additional micelle particles of resveratrol introduce heterogeneity into the system, causing interference with the measurement of light scattering intensity.

The results demonstrate that the binding of resveratrol to CFNPs can be controlled and precise. However, it was observed that exceeding a critical concentration of resveratrol can negatively impact the stability of the colloidal system. This suggests that there is a delicate balance between the beneficial binding of resveratrol and its potential destabilizing effect at higher concentrations.

### Encapsulation properties and physical stability of CFNPs-Res

Composed of proteoglycans and lipids, CFNPs are expected have abundant hydrophilic and hydrophobic regions to bind and encapsulate bioactive compounds. As depicted in Fig. 2D, G, CFNPs exhibited high encapsulation ability for resveratrol. The maximum encapsulation efficiency (70.8 ± 0.9%) was observed at the initial resveratrol concentration (10 µg/mL), followed by a decrease with the increasing resveratrol concentration. Meanwhile, the increasing resveratrol concentration resulted in a higher loading capacity and a maximum of 2.4 ± 0.01% reached at a resveratrol concentration of 150 µg/mL with an encapsulation efficiency of 59.3 ± 0.9% at this point. This suggests the resveratrol concentration plays an integral role in constructing ideal CFNPs-Res with a high loading capacity, which clearly confirmed the results of the size, PDI and ζ-potential exhibited beforehand.

To verify the effects of the physical removal protocol for free resveratrol on the stability of CFNPs-Res, centrifugation and membrane filtration were employed, respectively. The results have shown that both the encapsulation efficiency (EE) and loading capacity (LC) of CFNPs with varying resveratrol concentrations were slightly higher when samples were treated with centrifugation compared to membrane filtration. However, a significant difference was observed when the resveratrol concentration exceeded 150 µg/mL.

Protein-based systems, especially protein-polysaccharide complexes, have attracted extensive attention among the nano-delivery system due to their ability to effectively enhance the loading capacity, water-solubility, and stability of bioactive compounds^23^. The interaction of various proteins such as gelatin, zein, casein, and polysaccharides such as hyaluronic acid, pectin is systematically studied and employed to fabricated into the nanocarriers^24,25^. For example, the antisolvent precipitation method^26^ was used to expose the hydrophobic regions of the protein to form protein nanoparticles, while adding resveratrol to enhance the binding efficiency between resveratrol and protein nanoparticles. Another strategy involved the film-forming properties of proteins, which was utilized to generate microencapsulation and nanoencapsulation of bioactive ingredients using specific techniques like electrospray^27^.

The nanostructure of CFNPs is mainly formed by proteoglycans, which are integrated with biological lipid molecules, resulting in acid-base and high-temperature stability. CFNPs have demonstrated an encapsulation efficiency of 60% to 70% for resveratrol, which is consistent with the results reported in some other studies^28^.

This suggests that CFNPs have a noteworthy encapsulation efficiency for resveratrol and can be further optimized. Although CFNPs have been previously shown to successfully load bioactive compounds such as water-soluble ornithine, and hydrophobic phytosterols, the limited content of these compounds in clam may hinder their bioaccessibility in CFNPs. Consequently, the loading behaviour of CFNPs for these compounds may not fully reveal the potential of CFNPs as nanocarriers.

In general, smaller nanoparticles have a larger surface area, allowing for better dispersion of bioactive compounds and the increased bioavailability^29^. Compared to other reported nanoparticles^30^ with similar encapsulation efficiency, CFNPs-Res have consistently demonstrated particle sizes of less than 80 nm, while others often exceed 200 nm. The smaller size of CFNPs-Res suggests an increased potential for contact with the intestinal mucus layer, which may enhance absorption and delivery compared to other nanoparticles that often exceed 200 nm in size.

Although both centrifugation and membrane filtration are physical treatments, the filtration might have a stronger impact on the nanostructure of CFNPs based on the results and could potentially lead to the destruction. Recent studies have further investigated purification methods for nanoparticles, with membrane fractionation and centrifugation being among the most promising approaches. These results were consistent with those reported by Nishan et al. ^31^. which have shown that membrane fractionation resulted in smaller sized nanoparticles. As a result, centrifugation was chosen as the preferred protocol for removing free resveratrol in subsequent research.

### Fluorescence spectroscopy

**Fig. 3:**
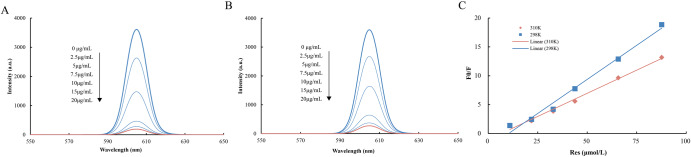
Fluorescence spectra. Fluorescence spectra of CFNPs-Res with different concentrations at (**A**) 310 K, and (**B**) 298 K. **C** F0/F linear curve of CFNPs-Res with different concentrations at two temperatures.

Fluorescence spectroscopy is a widely utilized technique for investigating the interaction between bioactive compounds. It is well known that polyphenol flavonoids, such as resveratrol, possess a high affinity for proteins and other biomolecules, as they can form stable complexes through noncovalent interactions^32^. As depicted in Fig. 3, CFNPs exhibited a maximum emission wavelength of 605 nm in the absence of resveratrol. Upon addition of resveratrol to the CFNPs solution at 298 K or 310 K, with concentrations ranging from 0 to 20 μg/mL, a concentration-dependent decrease in the fluorescence intensity of CFNPs was observed, ultimately resulting in more than 90% of the fluorescence intensity being quenched by resveratrol. This indicated a strong binding interaction of resveratrol with CFNPs, which was consistent with the protein-Res behaviour observed in a previous study conducted by Fan et al. ^33^.

Fluorescence quenching mechanisms are generally classified based on the interaction between the fluorophore of a substance and the quencher. Here, CFNPs were the substance, resveratrol acted as the quencher, and Stern-Volmer equation was used to explore the type and mechanism of Res-induced fluorescence quenching of CFNPs. Quenching parameters were obtained at different temperatures from Stern-Volmer curves (Fig. 3C) and are summarized in Table 1. The quenching constants Ksv and Kq decreased with increasing temperature from 298 K to 310 K, suggesting static quenching as the dominant mechanism. The molecular quenching constants Kq measured at both temperatures were far greater than the maximum collisional quenching rate of 2.0×10^10^ L/(mol·s)^34^, further supporting static quenching mechanism. Thermodynamic parameters including enthalpy change (ΔH°), entropy change (ΔS°), and Gibbs free energy change (ΔG°), calculated using the Van’t Hoff equation, can provide insights into the intermolecular forces present between bioactive compounds and proteins. These parameters generally can reveal whether van der Waals interactions (ΔH°<0 and ΔS°<0), hydrophobic forces (ΔH°>0 and ΔS°>0), or hydrogen bonds (ΔH°<0 and ΔS°<0) are involved^35^. At 298 K and 310 K, both ΔH° and ΔS° were negative, indicating that the binding of resveratrol to CFNPs nanoparticles was primarily driven by hydrogen bonding (Table 2). Furthermore, as the temperature increased, the binding constant of resveratrol to CFNPs decreased by nearly one order of magnitude, from 10^9.014^ to 10^8.233^, and the number of binding sites decreases from 1.90 to 1.75, which was in line with the typical behaviour of weakening hydrogen bonding with increasing temperature.

**Table 1:** Quenching constants of the interaction between CFNPs and Res at 298 K and 310 K

T (K)	R^2^	K_q_ (x10^10^M^−1^s^−1^)	K_sv_ (L*mol^−1^)	n
298	0.9937	2361.77	236177	1.90
310	0.9943	1604.55	160455	1.75

**Table 2:** Thermodynamics and binding parameters of CFNPs-Res interaction

T (K)	Ka (M^−1^)	n	△H° (Jmol^−1^)	△S°(Hmol^−1^K^−1^)	ΔG°(Jmol^−1^)
298	10^9.014^	1.90	−115099.50	−206.73	−53493.99
310	10^8.233^	1.75	−115099.50	−219.78	−46967.79

Yang et al.^36^ discovered that the exposure of tyrosine residues of myofibrillar proteins from mussels facilitated the formation of hydrogen bonds between protein-water/protein interfaces, as they could act as strong hydrogen bond acceptors. CFNPs, a kind of protein-polysaccharide based nanoparticle derived from shellfish, was expected to contain abundant tyrosine residues, thus enabling the interaction with resveratrol. Furthermore, some previous reports^24,37^ revealed that hydrogen bonds and electrostatic interactions were the primary driving forces for the interaction between protein and phenolic molecules.

Additionally, resveratrol contains acrylate group, which has potential to bind with polysaccharides through non-covalent interactions, such as hydrogen bonds, hydrophobic interactions, and electrostatic interactions, which is highly responsive to temperature. While fluorescence spectroscopy verified a strong interaction between CFNPs and resveratrol and provided insight into the mechanism of action, further studies are needed to explore the molecular mechanisms underlying this interaction and the potential applications of CFNPs as delivery systems for other bioactive compounds.

### Antioxidative capacity of CFNPs-Res within three-day storage at room temperature (25 °C)

**Fig. 4:**
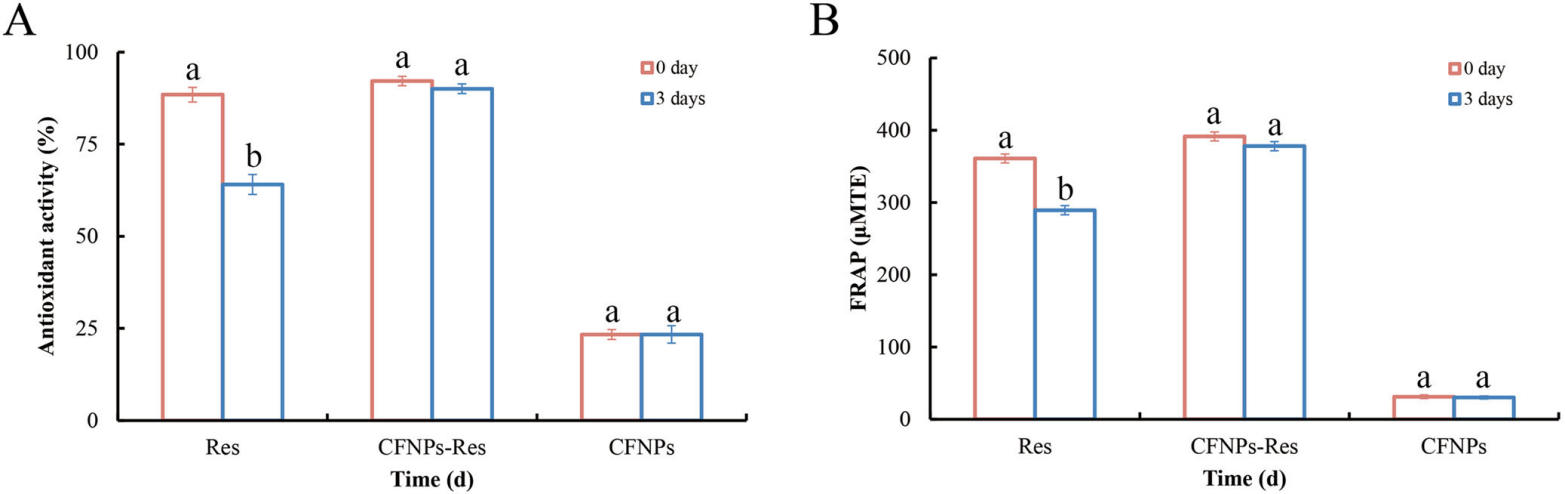
Antioxidative capacity of CFNPs-Res and free Res within three-day storage at room temperature. **A** ABTS assay and (**B**) FRAP assay at 0 d and 3 d. Mean values with different letters are significantly different between 0 and 3 days (*p* < 0.05) (*n* = 3). Error bars refer to standard deviation.

The antioxidative activity of resveratrol renders it highly attractive for the food pharmaceutical, and cosmetic industries, however, this activity can be damaged because of its sensitivity to environment conditions such as heat, light, and some chemicals^33^. To evaluate the protective effect of CFNPs encapsulation, the antioxidant activity of resveratrol was identified by ABTS and FRAP scavenging assay, respectively, before and after 3-day storage at room temperature in the dark. As shown in Fig. 4A, CFNPs alone was found to have a low antioxidative capacity (23.3 ± 1.3%), while CFNPs-Res and free resveratrol solution exhibited higher antioxidant activity with ABTS scavenging value of 92.1 ± 1.2% and 88.4 ± 1.9%, respectively. This indicated that the combination of resveratrol and CFNPs resulted in CFNPs-Res with an enhanced antioxidant capacity. Moreover, CFNPs-Res exhibited superior stability under room temperature conditions as compared to free resveratrol. Specifically, the ABTS scavenging value of CFNPs-Res after three days at room temperature remained over 90.0±1.3%, whereas the antioxidant capacity of free resveratrol significantly decreased to about 64.0 ± 2.7%. In Fig. 4B, it could be observed that the antioxidant (FRAP) value of resveratrol decreased significantly by 20% from 361.0 ± 5.1 µM TE to 289.3 ± 5.2 µM TE after being stored for three days. In contrast, CFNPs-Res exhibited only a small decrease in the FRAP value from 391.3 ± 6.0 µM TE to 377.9 ± 6.3 µM TE (*p* > 0.05), indicating a higher stability of CFNPs-Res compared to resveratrol alone.

These results suggested that the stability of resveratrol was effectively improved when combined with CFNPs, resulting in preservation of most of the antioxidant activity. These findings were consistent with the previous report by Yang et al.^28^. which indicated that nano-encapsulation strategy enabled to retain the activity of resveratrol. The protection of resveratrol by CFNPs may be attributed to the strong interaction between CFNPs and resveratrol, as evidenced by fluorescence spectroscopy, which leads to the formation of a compact nanoparticle structure that is highly resistant to oxidation from water-soluble oxygen promoters such as free radicals, dissolved oxygen, and some metals^33^.

### Bound resveratrol of wine measurement

Bioactive compounds in food can affect their functionality in various ways, including through their dosage and the way they are dispersed within the food matrix. To investigate the various forms of resveratrol in wine, the percentage of bound resveratrol in three selected wines was determined using ultrafiltration membrane. Additionally, the colloidal properties of the wines were analysed with DLS, too. The results revealed that varied between 1.11 and 1.25 µg/mL, with the percentage of bound resveratrol in the three wines ranging from 7.3 to 9.1%. The measured resveratrol content in the wines fell within the previously reported range^38^. Although the intensity of light scattering remained relatively stable, there was no discernible particle size distribution, as indicated by the large errors observed in particle size and PDI measurements, suggesting that the content of supramolecular aggregates was relatively low, and that most of resveratrol existed in its free form.

Wine is acknowledged for its elevated polyphenol levels, comprising anthocyanins, proanthocyanins, and polysaccharides^39^, that have the capability to interact and form colloidal aggregates within the wine matrix. Previous research by Suo et al.^40^. has also demonstrated the presence of high-molecular-weight polyphenolic complexes in red wine that can help improve metabolic dysregulation induced by a high-fat diet. It means the possibility of the bound resveratrol. This raises the possibility that bound resveratrol may be of greater importance to nutritional health than free resveratrol.

### In vitro release profiles of resveratrol

To simulate physiological fluidic condition in the body, the release profiles of resveratrol from CFNPs-Res, free resveratrol solution, and wines were studied in PBS buffer (pH 7.4).

**Fig. 5:**
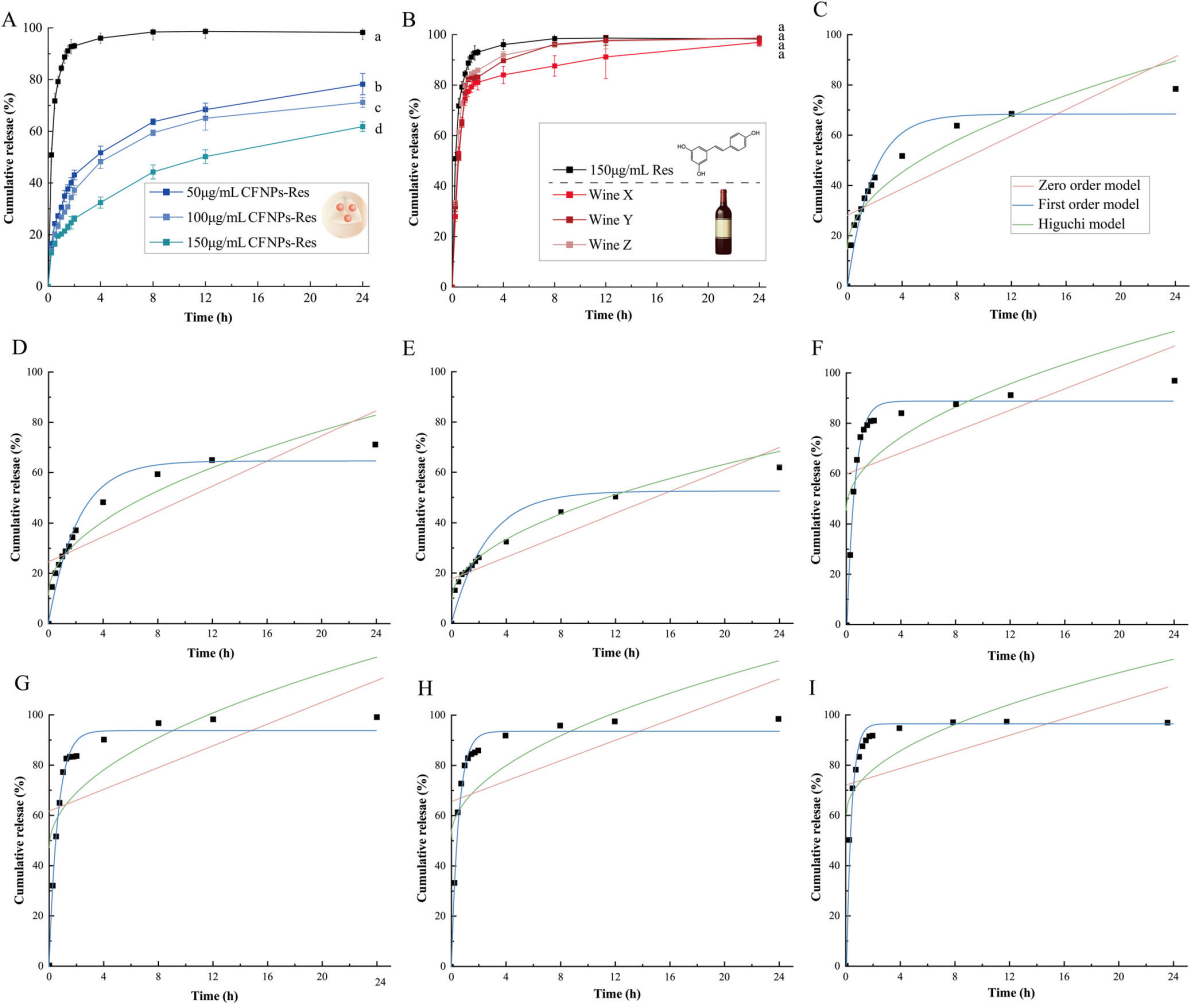
In vitro release curve of samples within PBS. **A** Release curves of 50 µg/mL CFNPs-Res, 100 µg/mL CFNPs-Res, 150 µg/mL CFNPs-Res and free Res. **B** Free Res and the three selected wines. **C** Fitting models of release curves of 50 µg/mL CFNPs-Res, (**D**)100 µg/mL CFNPs-Res, (**E**)150 µg/mL CFNPs-Res, (**F**) Wine X, (**G**) Wine Y, (**H**) Wine Z, and (**I**) 150 µg/mL free Res. Three fitting models including zero order, first order and Higuchi model were employed. Mean values with different letters are significantly different (*p* < 0.05) (*n* = 3). Error bars refer to standard deviation.

As depicted in Fig. 5B, there was a burst release within the initial hour for the free resveratrol solution and three wines. At the end of the forth hour, the release rates was nearly 90% for Wine Y and Wine Z, while that of Wine X was around 80%. After the initial period, the cumulative release rate reached a plateau, and the release curves became linear. In contrast, resveratrol loaded in CFNPs exhibited a slower release behaviour (Fig. 5A). Within 4 h, the cumulative release rate of CFNPs-Res was between 30 and 50%, and no burst release was observed. Furthermore, compared to the 100 µg/mL and 50 µg/mL CFNPs-Res nano-systems, the 150 µg/mL CFNPs-Res showed a more pronounced sustained release effect for resveratrol. These results were consistent with the colloidal properties and loading behaviour observed in Fig. 2. Thus, it can be concluded that CFNPs have the potential to serve as a platform for the controlled release of bioactive agents such as resveratrol.

Three common kinetic models (Fig. 5C–I), including zero-order, first-order, Higuchi’s matrix, were employed to fit the release kinetics of resveratrol from free resveratrol solution, CFNPs-Res and wines, respectively. The kinetic parameters of models were calculated and presented in Table 3. The results showed that the in vitro release kinetics of CFNPs-Res was well fitted to both first order and Higuchi’s matrix with R-squared just close to 0.9. Moreover, the R-squared value of the Higuchi model for the 150 µg/mL CFNPs-Res was over 0.97. In contrast, the release kinetics of free resveratrol and wines were fitted to the first-order model only. The first-order model was used to calculate the slope a1, which represents the relative release capacity. The results showed that the relative release capacity followed a specific order: 150 µg/mL free Res > Wine Y > Wine Z > Wine X, while 50 µg/mL CFNPs-Res > 100 µg/mL CFNPs-Res > 150 µg/mL CFNPs-Res. The Res release profiles of wines were consistent with the bound resveratrol proportion in wines (Table 4). These findings suggest that nanoaggregates, whether in wines or CFNPs-Res, play a critical role in the release kinetics of resveratrol.

**Table 3:** Fitting models of release kinetic parameters of CFNPs- Res, free Res, and wines

Sample/ Release kinetics		Zero order		First order		Higuchi	
		a_0_	b_0_	a_1_	b_1_	a_H_	b_H_
50 µg/mL CFNPs-Res	Equations	2.62	28.22	68.29	0.55	15.20	14.68
	R^2^	0.68224		0.93895		0.90124	
100 µg/mL CFNPs-Res	Equations	2.51	24.35	64.66	0.46	14.57	11.45
	R^2^	0.69691		0.95414		0.91303	
150 µg/mL CFNPs-Res	Equations	2.17	17.64	52.21	0.38	12.01	7.45
	R^2^	0.81535		0.88234		0.97085	
150 µg/mL Res	Equations	1.65	73.02	95.10	2.67	12.52	59.69
	R^2^	0.16718		0.98881		0.37638	
Wine X	Equations	2.12	59.90	88.51	1.69	14.48	45.40
	R^2^	0.27808		0.98304		0.50832	
Wine Y	Equations	2.23	62.37	93.82	1.55	15.45	46.78
	R^2^	0.27797		0.98599		0.52111	
Wine Z	Equations	2.03	65.70	93.33	1.89	14.40	50.93
	R^2^	0.23768		0.98525		0.4686

**Table 4:** Bound rate of resveratrol in wines

	Before UF (µg/mL)	After UF (µg/mL)	Bound rate (%)
Wine X	1.115 ± 0.010	0.101 ± 0.011	9.1
Wine Y	1.251 ± 0.006	0.099 ± 0.008	7.9
Wine Z	1.228 ± 0.007	0.090 ± 0.007	7.3

### Bioaccessibility under simulated in vitro gastrointestinal digestion

**Fig. 6:**
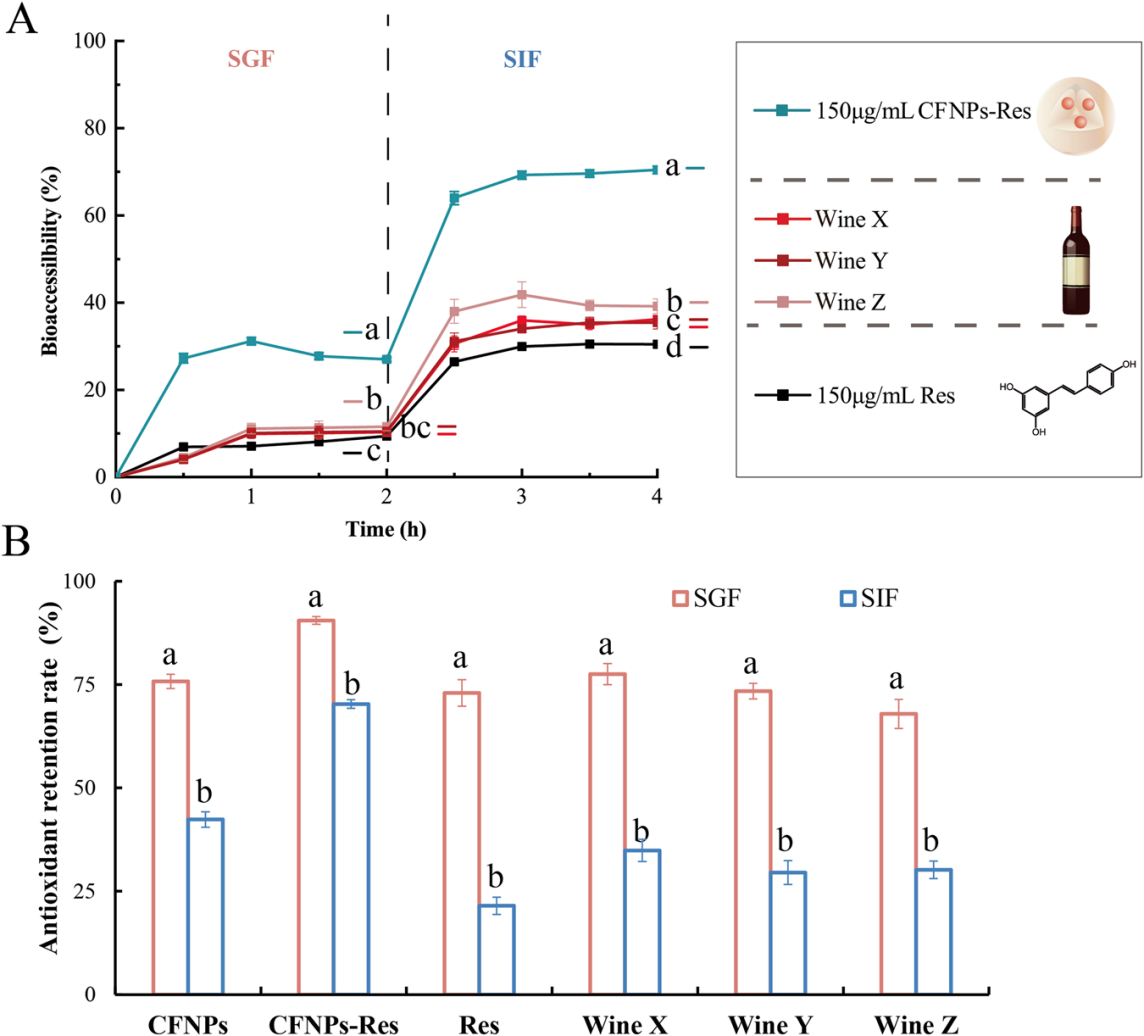
The bioavailability of samples in vitro gastrointestinal digestion. **A** The bioaccessibility of Res from free Res solution, CFNPs-Res and wines in vitro gastrointestinal digestion. **B** The retention of antioxidant capacity of free Res solution, CFNPs-Res and wines under simulated in vitro gastrointestinal digestion (*p* < 0.05) (*n* = 3). Error bars refer to standard deviation.

The release kinetics of bioaccessibility of free resveratrol, CFNPs-Res and wines under simulated gastrointestinal conditions were determined, respectively. Figure 6A shows that CFNPs-Res remained chemically stable during both gastric and intestinal digestion.

During the first half-hour of the SGF stage, the amount of resveratrol released from CFNPs-Res increased rapidly and fluctuated slightly during the subsequent 1.5 h. By the end of SGF stage, the release amount reached 26.9 ± 0.4%, which was like the amount released in the PBS buffer during the same period of time. In contrast, resveratrol in the other samples was slowly released during the SGF stage, with only about 10% released by the end of the SGF stage. But when exposed to SIF at pH 6.8, a sudden increase in resveratrol release could be observed in all samples. At the end of SIF, the resveratrol release of CFNPs-Res was up to 70.4 ± 0.8%, while those of the other samples (free resveratrol and wines) were 30–40%.

### Antioxidative capacity under simulated in vitro gastrointestinal digestion

Upon completing the assessment of the in vitro antioxidant capacity of freshly formulated and 3-day stored resveratrol loaded CFNPs, it was imperative to evaluate the antioxidant capacity under simulated in vitro gastrointestinal digestion. This approach was more realistic and representative of the way they function inside the human body. As demonstrated in Fig. 6, the antioxidant capacity of all samples, except for CFNPs-Res, decreased significantly after treatment with SGF for 120 min. The retention rate of antioxidant capacity for CFNPs-Res still exceeded 90% (with 90.5 ± 0.9%), while the values of other samples decreased to around 75%. Notably, the free resveratrol solution has shown the highest decrease in antioxidant capacity, by approximately 30%. After exposure to SIF for 120 min, the antioxidant activity of all samples decreased rapidly. The antioxidant retention rates of free resveratrol, CFNPs and CFNPs-Res solution were 21.4 ± 2.0%, 42.3 ± 1.8% and 70.3 ± 1.0%, respectively, indicating that CFNPs-Res played an effective role in protecting resveratrol during digestion.

Resveratrol is known to be highly sensitive to light, heat, and metabolic enzymes. In line with our findings, Huang et al.^30^. reported that the antioxidant activity of free resveratrol would be mostly lost after simulated digestion. The Res-loaded nanoparticles were found to be effective in maintaining the antioxidant activity of resveratrol, owing to the possible enhancement of resveratrol water solubility and physicochemical stability by nanostructures, leading to enhanced bioavailability and bioaccessibility. On top of it, the physical barrier effect of CFNPs was likely to have played a significant role in the protection and controlled release of resveratrol in buffer system and in vitro gastrointestinal digestion.

Previous studies^41^ have reported that CFNPs exhibit remarkable tolerance to harsh conditions such as high temperatures and extreme acidity and were also responsive to metabolic enzymes such as amylase. The rapid increase in bioaccessibility of resveratrol and the decline in the antioxidant activity of CFNPs and CFNPs-Res after simulated intestinal digestion were probably attributed to the hydrolytic breakdown of the proteoglycan scaffold of CFNPs.

Of note, digestion in the human body, however, involves not only the reactions between compounds and digestive enzymes but the interplay between compounds and various cells, such as those found in mucosa of digestive tract: epithelial cells, immune cells, and intestinal villus cells. Ke et al. ^42^. conducted and found that colloidal particles isolated from bone soup could be rapidly ingested by gastrointestinal macrophages, which might occur before the particles were completely digested by the digestive fluid, effectively regulating the oxidative stress state of macrophages. In addition, extracellular vesicles from milk^43^ and even some fruits^44,45^, which have been extensively studied in recent years, have been elucidated to deliver functional factors in the body despite harsh digestive environments. Anyway, CFNPs might have the potential to deliver other bioactive compounds, such as curcumin and quercetin, with similar physicochemical properties as resveratrol.

Considering the low bioavailability of resveratrol, a daily intake of one gram of this compound has been recommended to achieve its therapeutic efficacy^38^. This dose was previously believed to be achievable only through purified dietary supplements rather than traditional foods. However, with the promotion of nanocarrier-based technologies, the bioavailability of resveratrol has been significantly improved, potentially leading to a significant change in the recommended daily dose. Moreover, the binding of resveratrol to CFNPs and the resulting improved bioavailability suggest the possibility of nanocarriers in traditional foods enhancing the absorption of bioactive compounds, such as steamed clam with wine. These findings provide novel insights into the nutritional mechanisms and the potential application of traditional foods, novel functional foods, as well as pharmaceutical agents.

## Discussion

We demonstrated that nanoparticles in freshwater clam soup can carry resveratrol and render it more stable and bioavailable. The interaction between CFNPs and resveratrol generated CFNPs-Res with a satisfactory encapsulation efficiency and a particle size below 100 nm. CFNPs-Res has shown excellent stability in various conditions, including room-temperature storage, physical treatment, and the improved bioaccessibility and oxidative stability in the simulated gastrointestinal digestion. A controlled release of resveratrol from CFNPs-Res in PBS buffer imitating body fluids was also demonstrated.

In addition, the resveratrol bound to the nanoaggregates in wines exhibited differences compared to the free resveratrol, in terms of release kinetics and stability during in vitro digestion, echoing the assumption that the food derived nanoaggregates may facilitate the delivery of micronutrients or bioactive compounds.

Protein-polysaccharide hybrid nanocarriers have been widely designed and prepared. However, the fabrication is time-consuming and costly, relying on multiple non-covalent bonding between isolated ingredients (e.g., chitosan, grain proteins) or the covalent conjugation, such as glycation of caseinate with dextran. Here, we propose the possibility that the self-assemble nanoparticles derived from cooking the clams could be used to carry bioactive compounds.

We suggest that since nanoparticles are found in clam soup, they may also be presented in other food items with similar characteristics, and they too could potentially enhance the absorption of micronutrients and antioxidants in everyday cuisine, especially among populations facing micronutrient deficiencies. Due to the nature of their food origin, this approach of delivering micronutrients and bioactive components would have much less safety and ethical concerns^46^.

## Methods

### Materials

Resveratrol (purityå 97%, CAS 501–36–0), ABTS (2,2’-Azino-bis (3-ethylbenzthiazoline-6-sulphonic acid)) (CAS 30931–67–0) for ABTS antioxidant assay, TPTZ (2,4,6-tris(2-pyridyl)-s-triazine)(CAS 3682–35–7) for ferric reducing antioxidant power (FRAP) assay, pepsin from porcine gastric mucosa (CAS 9001–75–6), pancreatin from porcine pancreas (4X UPS, CAS 8049–47–6), bile extract porcine (CAS 8008–63–7) were purchased from (Sigma-Aldrich Co. Ltd, USA). Wines including Pinot Noir (Wine X), Merlot (Wine Y) and Cabernet Sauvignon (Wine Z) produced in Chile were purchased online. Freshwater clam was donated by Lichuan Fishery (Hualien, Taiwan, China). The deionized water was obtained using the Millipore Milli-Q water purification system (Millipore, Bedford, MA, USA), and ultrafiltration tubes (100 kDa) were purchased from (Millipore, USA). All other analytical grade chemicals and reagents were obtained from Sinopharm Chemical Reagent Co., Ltd.

### Preparation of freshwater clam soup and isolation of CFNPs from soup

According to a previously established protocol^15^ with slight modifications, freshwater clams were cultivated in cool water for a duration of 12 h. Subsequently, the clams were washed and cooked for 60 min with pure water in a 1:1 (w/v) ratio. The resultant soup was pooled together and subjected to centrifugation at 5000 ×*g* for 30 min to remove the solid residues. The supernatant was collected and preserved at a temperature of −20 °C until further analysis.

CFNPs were isolated using a well-established protocol^15^ with some modifications. The clam soup stored at −80 °C was removed and thawed at room temperature for stock. The stock soup was shaken and centrifuged at a high speed (5000 × *g* for 10 min) and the supernatant was extracted. The supernatant was further processed by ultrafiltration through a 100 kDa membrane at 4500 × *g* for 30 min, with the retained particles being washed twice with Milli-Q water and centrifuged again. The final retained pellet was resuspended in an equal volume of Milli-Q water to obtain the CFNPs solution which were stored at −20 °C for further use.

### Preparation of CFNPs-Res

Stock resveratrol solution (resveratrol dissolved in anhydrous ethanol) was added dropwise to CFNPs solution (3.5 mg/mL) to obtain a final concentration of 10, 20, 30, 50, 100, 150, and 200 μg/mL. The mixture was continuously and homogeneously stirred using a magnetic stirrer at room temperature for 30 min under dark conditions. Following the unbound resveratrol was removed from the system with centrifuge at a speed of 12,000 × *g* for 20 min or with a 0.22 μm cellulose acetate membrane for aqueous phase.

### Characterisation of CFNPs-Res

All samples were subjected to colloidal characterization including size, ζ-potential, and polydispersity index (PDI) using a Malvern instrument (Zetasizer Nano-ZS, Malvern Instruments Ltd, UK) at 25 °C. Each sample was determined at least three times prior to data analysis. Then the morphology of samples was imaged using a JEOL JEM-1230 transmission electron microscope (TEM, Japan). The samples were first placed on copper grids with a mesh size of 230 and stained with phosphotungstic acid. Subsequently, they were observed under the TEM at an acceleration voltage of 80 kV.*2.5*.

### Encapsulation properties and stability of CFNPs under physical treatment

The encapsulation efficiency (EE) and loading capacity (LC) of resveratrol loaded into CFNPs were calculated using Eqs. (1), (2), respectively.

1 $${\rm{Encapsulation\; efficiency}}=\frac{{total\; Res}-{free\; Res}}{{total\; Res\; input}}\times 100 \%$$

2 $${\rm{Loading\; capacity}}=\frac{{total\; Res}-{free\; Res}}{{total\; mass\; of\; nanocomplexes}}\times 100 \%$$

A UV–Vis spectrophotometer (UV-5100, Hitachi, Japan) was used to determine the resveratrol content at room temperature and the absorbance was detected at the maximum wavelength of 305 nm. A calibration curve of the absorbance value versus the resveratrol concentration was plotted and fitted with a linear function (R^2^ = 0.9993, 1–20 μg/mL). The equation of the calibration curve was y= 0.2222x-0.014 (y is the resveratrol concentration, μg/mL, and x is the absorbance value).

The stability of CFNPs under physical treatment was evaluated by subjecting them to two common procedures: centrifugation and membrane filtration. CFNPs were either centrifuged at a speed of 12,000 × *g* for 20 min or filtered through a 0.22 μm aqueous membrane, and then the content of resveratrol retained was determined.

### Fluorescence spectral analysis

The fluorescence spectrophotometric method was applied to analyse the interaction between resveratrol and CFNPs. The fluorescence was measured with an infrared spectrometer (F-4700, Hitachi, Hitachi High-Technologies, Ltd., Japan) at 298 K and 310 K, respectively, by slowly adding different concentrations of resveratrol to the CFNPs solution to achieve a final concentration of 2.5–20 μg/mL of resveratrol. The excitation wavelength was 302 nm, and the emission spectrum was scanned at the full wavelength in the range 200–900 nm, where the slit width for both emission and excitation was set to 5 nm. The fluorescence quenching data was analysed according to the Stern-Volmer Eq. (3).

3 $$\frac{{F}_{0}}{F}=1+{k}_{q}{\tau }_{0}\left[Q\right]=1+{K}_{{sv}}[Q]$$

In Eq. (3), F_0_ and F represent the fluorescence intensity of the CFNPs in the system without and with the addition of resveratrol, respectively. Kq is the molecular quenching constant. τ_0_ is the fluorescence lifetime in the absence of quencher (in this experiment, resveratrol acts as a quencher), typically 10^–8^ s^47^. Ksv is the Stern-Volmer bursting constant. [Q] is the concentration of the bursting agent. The binding constant and the number of binding sites were analysed by means of the double logarithmic Eq. (4).

4 $${\mathrm{lg}}\frac{{F}_{0}-F}{F}={\mathrm{lg}}{K}_{a}+{nlg}[Q]$$

In Eq. (4), n is the number of binding sites and Ka is the binding constant.

### Thermodynamic analysis

Generally, thermodynamic parameters including the enthalpy (ΔH°), entropy (ΔS°) and the change in Gibbs free energy (ΔG°) can be calculated based on Van’t Hoff equation. Equations (5), (6) show the Van’t Hoff equation,

5 $${\mathrm{ln}}\left(\frac{{\rm{Ka}}2}{{\rm{Ka}}1}\right)=\left(\frac{1}{{\rm{T}}1}-\frac{2}{{\rm{T}}2}\right)\times \frac{\Delta {\rm{H}}^{\circ}}{{\rm{R}}}$$

6 $$\Delta {\rm{G}}^{\circ} =-{\rm{RT}}{\mathrm{ln}}{\rm{Ka}}=\Delta {\rm{H}}^{\circ} -{\rm{T}}\Delta {\rm{S}}^{\circ}$$

where R is the gas constant (8.314 Jmol^−1^ K ^−1^) and T is the experimental temperature.

### Antioxidant activity stability of CFNPs-Res under 3-day storage

The antioxidant activity stability of 150 µg/mL CFNPs-Res, and free resveratrol solution (150 µg/mL) under room temperature were assessed by ABTS and FRAP assay^48^, while a CFNPs solution was used as a control. The antioxidant capacity of the samples was determined on day 0 and day 3 under dark conditions to evaluate the potential protective effects of the CFNPs on the activity of resveratrol.

#### ATBS assay

Specifically, to prepare the ABTS solution, a potassium persulfate solution (2.6 mM) was mixed with an ABTS solution (7.4 mM) at a 1:1 (v/v) ratio and left in the dark at 25◦C for 12–16 h. The resulting ABTS radical cationic solution was diluted to obtain an absorbance of 0.70 ± 0.02 at 734 nm. Then, 40 μL of the test sample was mixed with 4.0 mL of the ABTS solution in the dark for 5 min, and the absorbance was measured at 734 nm using a UV–Vis spectrophotometer (UV-5100, Hitachi, Japan). ABTS antioxidant activity was calculated using the following Eq. (7).

7 $${\rm{Antioxidant\; activity}}\left. ( \% \right)=\frac{{A}_{0}-{A}_{1}}{{A}_{0}}\times 100 \%$$

Here, A_0_ and A_1_ are the absorbance values of control solution and samples at 734 nm, respectively.

#### FRAP assay

The working FRAP reagent was prepared by mixing 10 mL of acetate buffer (0.3 M, pH 3.6), 1 mL of tripyridyltriazine solution (TPTZ, 10 mM) prepared in HCl (40 mM) and 1 mL of ferric chloride solution (FeCl_3_·6H_2_O, 20 mM). 10 mM FeSO_4_·7H_2_O solution was prepared and diluted to 0.15, 0.30, 0.60, 0.90, 1.20 and 1.50 mM as the standards for a calibration curve. An aliquot of dilute sample (5 μL) and FeSO4·7H_2_O standards (5 μL) were pipetted into a 96-well plate respectively, then 180 μL working FRAP reagent was added to each well and then incubated at 37 °C for 5 min. The distilled water (5 μL) was set as the control. The absorbance at 593 nm was measured three duplicates with FlexStation 3 Plate Reader (Molecular Devices, Sunnyvale, CA, USA) at 37 °C. FRAP antioxidant activity was calculated using the following Eq. (8) and expressed with Trolox equivalent (TE).

8 $${\rm{FRAP\; value}}\left. ({\rm{TE}}\right)=\frac{{A}_{1}\,-\,{A}_{0}}{{slope\; of\; standard\; curve}}\times 100 \%$$

Here, A_0_ and A_1_ are the absorbance values of control solution and samples at 593 nm, respectively. The standard curve for FRAP value was established, with an equation of y = 0.0026x + 0.0585 (R² = 0.9972), where x represents the Trolox concentration and y represents the corresponding absorbance value.

### Measurement of bound resveratrol content in wines

Ultrafiltration was employed to isolate nanoaggregates from a mixture and to extract bound resveratrol from selected wines. Initially, the wines were centrifuged at 3000 g for 15 min to remove precipitates, and the resulting supernatant was then subjected to ultrafiltration (MW = 100 kDa cut-off) at 4500 g for 30 min. The retained substances were subsequently washed three times and dissolved in deionized water, and the bound resveratrol content was determined using a UV–Vis spectrophotometer (UV-5100, Hitachi, Japan).

### In vitro release within PBS

The in vitro release of resveratrol from both free resveratrol solution, CFNPs-Res and wines was assessed using a dialysis tube (8–14 kDa). Specifically, 8 mL of sample was loaded into the dialysis bag, which were then placed in 200 mL of 0.02 M, pH 7.4 PBS buffer in a dark environment. Magnetic stirring was applied at 37 °C under dark conditions, and aliquots of 3 mL were withdrawn at 0.25, 0.5, 0.75, 1, 1.25, 1.5, 1.75, 2, 4, 8, 12, 24 h, respectively, with equal volumes of fresh PBS buffer added immediately. The concentration of resveratrol in each sample was quantified using a UV-Vis spectrophotometer (UV-5100, Hitachi Co., Japan) at a wavelength of 305 nm.

To analyze the in vitro release data, three mathematical models were employed: zero-order model, first-order model, and the Higuchi model^49^.

Zero-order kinetics: M_t_/M_∞_=a_0_t+b_0_

First-order kinetics: M_t_/M_∞_ = a_1_ (1- exp(-b_1_t))

Higuchi: M_t_/M_∞_ = a_H_t^0.5^+ b_H_

Here, M_t_/M_∞_ is the cumulative drug release rate, a is the release rate constant, b represents the initial amount of resveratrol.

### The bioaccessibility under in vitro simulated gastrointestinal digestion

The in vitro bioaccessibility of resveratrol was determined as the fraction of resveratrol that was solubilized within mixed colloids in the gastrointestinal fluids according to Huang et al.^30^. The release of resveratrol from the different samples (150 µg/mL CFNPs-Res, 150 µg/mL free resveratrol solution, three selected wines) was measured during the gastric and small intestine phases with an interval of 0.5 h. At the end of intestine phase, the samples were centrifuged at 5000 × *g* for 30 min, the supernatant was collected, and filtered using a 0.45 μm filter membrane. The resveratrol content in the supernatant was measured spectrophotometrically at 306 nm after being diluted in DMSO. The in vitro bioaccessibility of resveratrol was calculated using the following Eq. (9).

9 $${\rm{Bioaccessibility}}=\frac{{mass\; of\; solubilized\; Res}}{{mass\; of\; Res\; before\; digestion}}\times 100 \%$$

#### Simulated gastric phase

10 mL of sample was added to 7.5 mL of SGF stock solution. Next, 1.6 mL of pepsin with an enzyme activity of 25,000 U/mL was added, followed by 5 μL of 0.3 M CaCl_2_ solution and 0.2 mL of 1 M HCl solution. The reaction was carried out at 37 °C for 2 h while adjusting the pH of each system to 3 and adding 695 μL of water.

#### Simulated intestinal phase

20 mL samples which completed the whole gastric digestion phase was removed and 11 mL of SIF stock solution, 5 mL of trypsin, 2.5 mL of 160 mM bile salts, 40 μL of 0.3 M CaCl_2_, and a small amount of 1 M NaOH were added to adjust the pH to 7. Finally, 1.31 mL of water was added to fix the volume, and the reaction was carried out at 37 °C for 2 h.

### Antioxidant capacity under in vitro simulated gastrointestinal digestion

The samples were measured by ABTS assay after incubating for 120 min in SGF and for 120 min in SIF. The retention rates of antioxidant in different samples were calculated with Eq. (10):

10 $$\begin{array}{l}{\rm{Antioxidant}}\; {\rm{retention}}\,\left( \% \right)=\\ \dfrac{{{\rm{Antioxidant}}\; {\rm{activity}}}_{0}-{A{\rm{ntioxidant}}\; {\rm{activity}}}_{1}}{{{\rm{Antioxidant}}\; {\rm{activity}}}_{0}}\times 100 \%\end{array}$$

Here, antioxidant activity_0_ and antioxidant activity_1_ are the antioxidant activity before digestion and after (gastric or intestinal) digestion, respectively.

### Statistical analysis

All experiments were conducted at least three times to ensure reproducibility and reliability. The collected data were analyzed using SPSS software (IBM SPSS Statistic 26.0). One-way ANOVA with Tukey’s test and multiple comparison post hoc tests (LSD in equal variances and Tamhane’s with unequal variances) were performed to assess significant differences among samples at a *p*-level of 0.05.

## Data Availability

The authors declare that the data supporting the findings of this study are available within the article.
